# One-pot hydrothermal synthesis of Mn_3_O_4_ nanorods grown on Ni foam for high performance supercapacitor applications

**DOI:** 10.1186/1556-276X-8-535

**Published:** 2013-12-19

**Authors:** Dongwei Li, Fanhui Meng, Xiuling Yan, Lishan Yang, Hua Heng, Ye Zhu

**Affiliations:** 1 School of Chemistry and Chemical Engineering, Shandong University, Jinan 250100, China; 2 School of Chemistry and Bioscience, Ili Normal University, Xinjiang 835000, China; 3 School of Chemistry and Chemical Engineering, Hunan Normal University, Changsha 410081, China

**Keywords:** Manganese oxide, Ni foam, Nanorod, Hydrothermal synthesis, Supercapacitor

## Abstract

Mn_3_O_4_/Ni foam composites were synthesized by a one-step hydrothermal method in an aqueous solution containing only Mn(NO_3_)_2_ and C_6_H_12_N_4_. It was found that Mn_3_O_4_ nanorods with lengths of 2 to 3 μm and diameters of 100 nm distributed on Ni foam homogeneously. Detailed reaction time-dependent morphological and component evolution was studied to understand the growth process of Mn_3_O_4_ nanorods. As cathode material for supercapacitors, Mn_3_O_4_ nanorods/composite exhibited superior supercapacitor performances with high specific capacitance (263 F · g^-1^ at 1A · g^-1^), which was more than 10 times higher than that of the Mn_3_O_4_/Ni plate. The enhanced supercapacitor performance was due to the porous architecture of the Ni foam which provides fast ion and electron transfer, large reaction surface area, and good conductivity.

## Background

Pseudocapacitors, based on reversible redox reactions at/near the surface of the electrode, represent one type of supercapacitors having the potential for high energy densities [1-3]. As is known, the excellent electrode should primarily meet the following key requirements: (1) a large number of electroactive sites, (2) high transport rates of both electrolyte ions and electrons, and (3) high electronic conductivity [4]. Among various pseudocapacitor electrode materials, RuO_2_ has been extensively studied because of its ultrahigh theoretical capacitance (2,000 F · g^-1^ in a wide potential window of 1.4 V), a nearly metallic electrical conductivity and excellent chemical stability [5]. However, RuO_2_ has the drawbacks of high cost and toxicity. Therefore, extensive efforts have been made to search for alternative materials, such as Ni, Co, or Mn-based oxides/hydroxides [6-9]. Because the energy density of a supercapacitor is proportional to the square of the cell voltage, the energy density of Ni- and Co-related materials is limited by the narrow potential window [10].

Mn_3_O_4_ is a potentially interesting electrode material for electrolytic supercapacitors due to its low cost, non-toxicity, environmental compatibility, and intrinsically high capacity [11,12]. However, the capacitance property of Mn_3_O_4_ has been rarely investigated because of its poor electronic conductivity. A common strategy with poor electronic conductors is to combine them into composites with conducting substrates such as nanoporous gold, various carbon materials, and Ni foam [13,14]. Ni foam, as a commercial material with high electronic conductivity and a desirable three-dimensional (3D) structure is widely used as the electrode substrate material [15,16]. It would not only reduce the diffusion resistance of electrolytes but also provide a large surface area for loading active material. There have been some reports on the synthesis of Ni- and Co-based oxides/hydroxides on Ni foam [17-20]. However, there are very few reports on the fabrication of Mn-based oxides/hydroxides on Ni foam, except for the MnO_2_/CNT/Ni foam electrode [21,22]. To the best of our knowledge, one-pot hydrothermal synthesis of Mn_3_O_4_ nanorods structures on Ni foam has not been reported.

Here, we report facile direct synthesis of Mn_3_O_4_ nanorods on Ni foam with diameters of about 100 nm and lengths of 2 to 3 μm via one-pot hydrothermal process, without any additional surfactant. The extraordinary redox activity of the Mn_3_O_4_/Ni foam composite is demonstrated in terms of pseudocapacitive performance. The effect of reaction time on the crystal growth mechanism and supercapacitor performance of the Mn_3_O_4_/Ni foam is well discussed.

## Methods

### Chemicals

Hexamethylene tetramine (C_6_H_12_N_4_) and Mn(NO_3_)_2_ (50%) solution were purchased from Shanghai Chemical Reagent Company (Shanghai, China), while Ni foam (5 g/100 cm^2^) was purchased from Changsha Liyuan New Material Co., Ltd. (Changsha, China). All reagents used in this experiment were of analytical grade without further purification. The Ni foam was immersed in concentrated hydrochloric acid for 10 min and then washed with acetone, ethanol, and distilled water several times before use.

### Synthesis of samples

In a typical procedure, 3 mL Mn(NO_3_)_2_ (50%) solution and 2 g C_6_H_12_N_4_ were dissolved in 17 mL distilled water. After vigorously stirring, the resulting solution and the pre-cleaned Ni foam were transferred into a Teflon-lined stainless autoclave. The autoclave was sealed at 120°C for 10 h and then cooled to room temperature naturally. The products were washed with distilled water several times, and finally dried in a vacuum desiccator at 50°C. The deposit weight of Mn_3_O_4_ was accurately determined by calculating the weight difference between the Ni foam coated with Mn_3_O_4_ after the hydrothermal process and the Ni foam before the hydrothermal process.

### Characterization

The morphology of samples was characterized by scanning electron microscopy (SEM, JEOL JSM-6700 F, Akishima-shi, Japan) at an accelerating voltage of 10 kV. The obtained samples were characterized by X-ray powder diffraction (XRD) on a Bruker D8 advanced X-ray diffractometer (Madison, WI, USA) with Cu Ka radiation (*λ* = 1.5418 Å) at a scan rate of 0.02° · s^-1^. Raman spectra were obtained using LabRAM HR UV/vis/near-IR spectrometer (Kyoto, Japan) with an argon-ion continuous-wave laser (514.5 nm) as the excitation source.

The electrochemical measurements were performed in a standard three-electrode cell on a CHI 760D potentiostat at room temperature, where 1 cm^2^ (1 × 1 cm) of the obtained composite was used as the working electrode, a Pt plate was chosen as the counter electrode and a saturated calomel electrode (SCE) was selected as the reference electrode. A 4-M NaOH solution was used as the electrolyte.

## Results and discussions

### Component characterization

**Figure 1:**
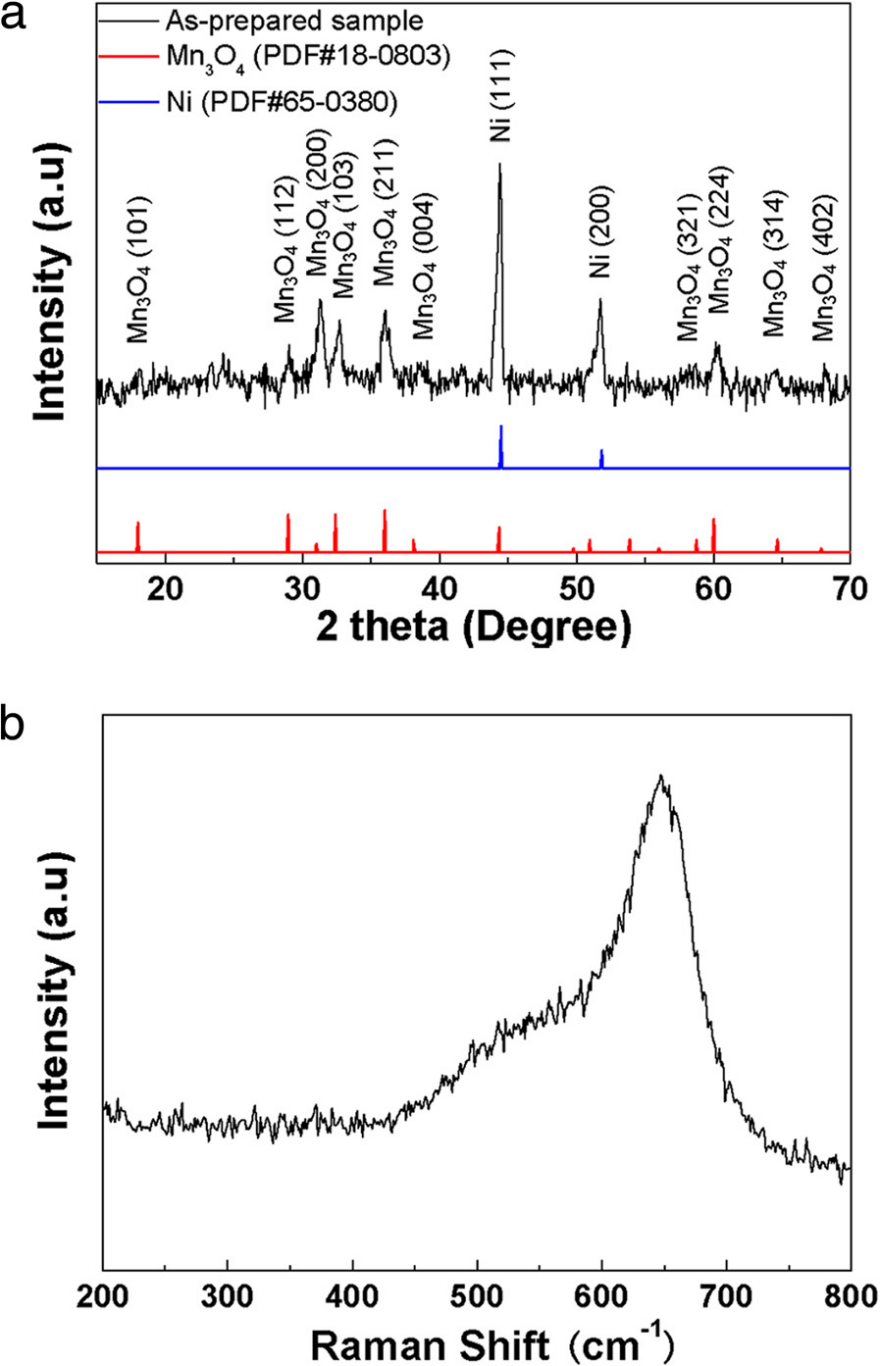
**XRD pattern (a) and Raman spectra (b) of Mn**_**3**_**O**_**4**_**/Ni foam composite.**

To examine the phase composition and structure of the samples, XRD analysis was carried out and the pattern is shown in Figure 1a. The as-prepared sample displays typical hausmannite Mn_3_O_4_ diffraction lines, which is in agreement with JCPDS card 18–0803. The peaks at around 44° and 52° are indexed to the Ni planes (111) and (200) of the Ni foam substrate, respectively. This result indicates that the utilized hydrothermal conditions are favorable for the formation of pure Mn_3_O_4_. Moreover, the XRD peaks are relatively broad, indicating that the crystals constituting the products are small in size. Raman spectra can be used to gain more information about structure (Figure 1b). Consistent with the XRD data, the peak at 652.3 cm^-1^ corresponding to the crystalline Mn_3_O_4_ structure are clearly observed [23].

### Morphology characterization

**Figure 2:**
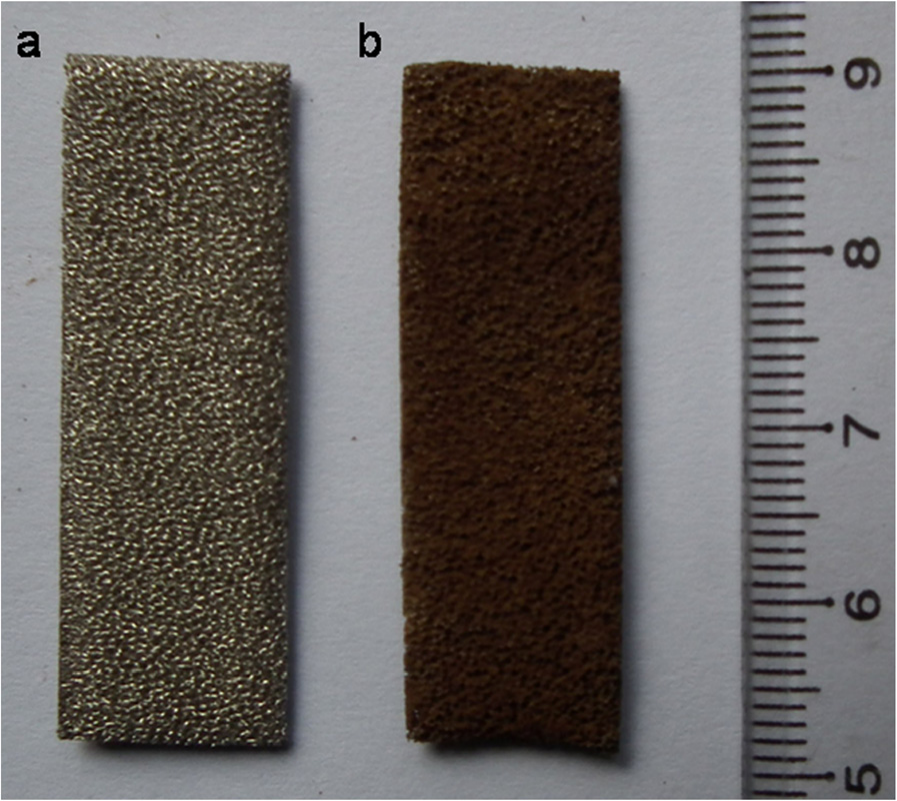
**Digital photographs of (a) the Ni foam and (b) Mn**_**3**_**O**_**4**_**/Ni foam composite.**

**Figure 3:**
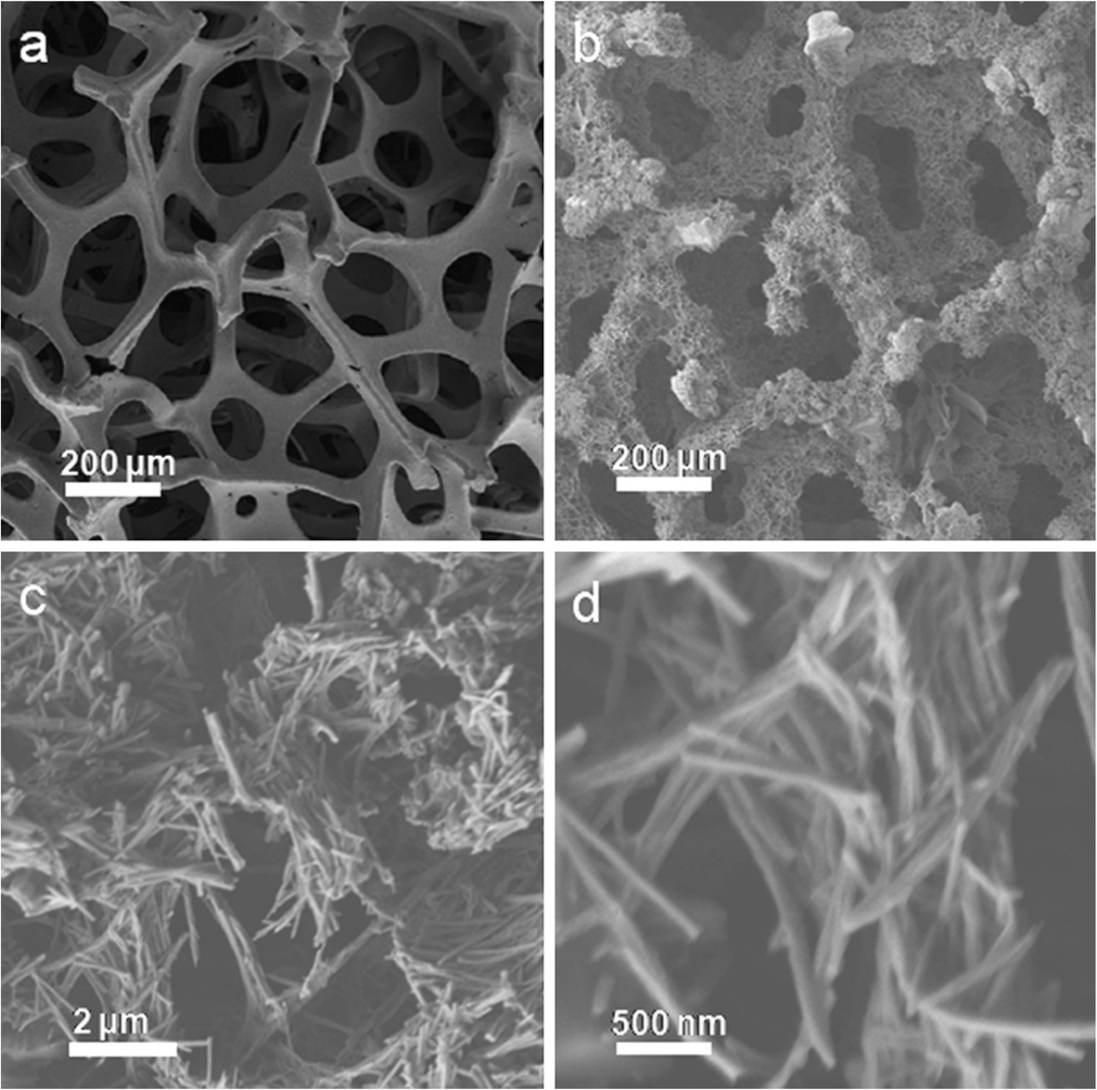
**SEM images of (a) the 3D structure of Ni foam and (b,c,d) Mn**_**3**_**O**_**4**_**/Ni foam composite with different magnifications.**

The photographs of the Ni foam (a) and the Mn_3_O_4_/Ni foam composite (b) are shown in Figure 2. The Ni foam turns to brown color after hydrothermal reaction, suggesting the formation of Mn_3_O_4_ on the Ni foam. The SEM image at low magnification shows that the pristine Ni foam has a 3D porous structure (Figure 3a). This porous skeleton of Ni foam would provide effective electrolyte accessible channels for ion transportation, and shorten the distance for ion diffusion. Figure 3b,c,d shows SEM images of the Mn_3_O_4_/Ni foam composite at different magnifications. These images show highly dense nanorods on Ni foam substrate. The individual nanorod is approximately 100 nm and approximately 2 to 3 μm in diameter and length, respectively, and the aspect ratio is greater than 20 in most cases.

### Electrochemical capacitance of Mn_3_O_4_/Ni foam electrode

**Figure 4:**
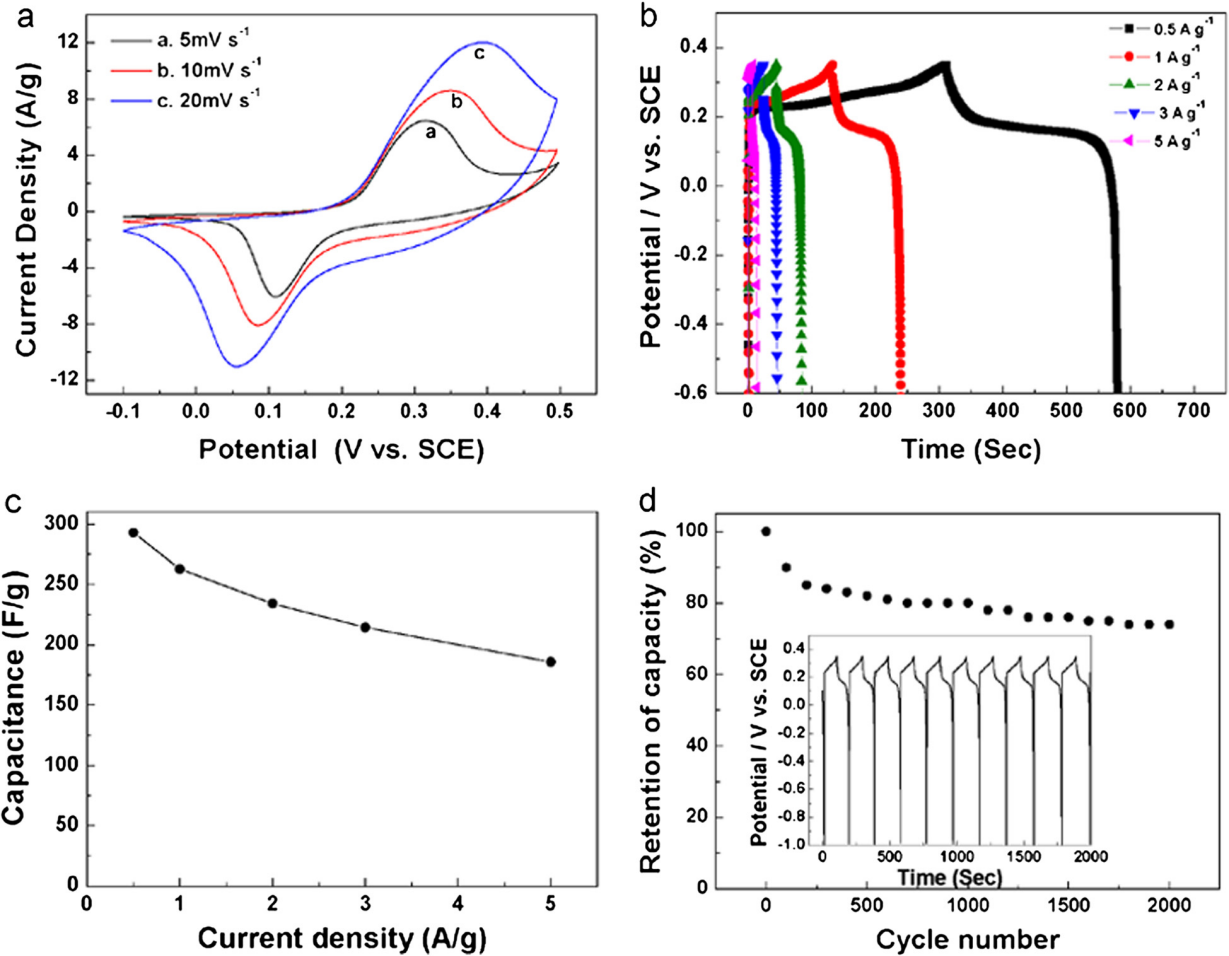
**CV and charging-discharging curves, corresponding specific capacitance, and capacitance retention of Mn**_**3**_**O**_**4**_**/Ni foam electrode. (a)** CV curves of the Mn_3_O_4_/Ni foam electrode at different scanning rates. **(b)** Charging-discharging curves of the Mn_3_O_4_/Ni foam electrode at different current densities. **(c)** The corresponding specific capacitance as a function of current density. **(d)** Capacitance retention of the Mn_3_O_4_/Ni foam electrode as a function of cycle number. The insert shows the charging-discharging profiles of the first ten cycles recorded with a current density of 1 A · g^-1^.

Cyclic voltammetry (CV) and galvanostatic charging-discharging measurements were performed to evaluate the electrochemical properties and quantify the specific capacitance of the Mn_3_O_4_/Ni foam composite. The CV curves of the composite at different scan rates between 5 and 20 mV · s^-1^ are presented in Figure 4a. Different from an ideal rectangular shape of the typical electrical double-layer capacitance, the redox reaction peaks indicate that the capacitance mainly results from the pseudocapacitive capacitance [24]. The pseudocapacitance arises from the reaction between the Mn^4+^ ions and NaOH electrolyte [25,26]. The peak current increases when the scan rate increases from 5 to 20 mV · s^–1^, while the anodic peaks shift toward the positive potential and cathodic peaks shift toward the negative potential, which demonstrates the quasi-reversible nature of the redox couples [27,28].

The charging-discharging curves of the Mn_3_O_4_/Ni foam were measured at various current densities (shown in Figure 4b). The specific capacitance was calculated according to the following equation:

C=i×ΔtΔV

where *C* (F · g^-1^) is the specific capacitance; *i* (A · g^-1^) is the discharge current density, *Δt* (s) is the discharge time, and *ΔV* (V) is the discharge potential range. The specific capacitance values of the Mn_3_O_4_/Ni foam composite evaluated from the discharge curves are 293, 263, 234, 214, and 186 F · g^-1^ at the current density of 0.5, 1, 2, 3, and 5 A · g^-1^, respectively (Figure 4c). The significant capacitance decrease with increasing discharge current density is likely to be caused by the increase of potential drop due to electrode resistance and the relatively insufficient Faradic redox reaction of the Mn_3_O_4_/Ni foam composite under higher discharge current densities. It is noteworthy that the specific capacitance of the as-prepared Mn_3_O_4_/Ni foam composite is higher than of the previously reported Mn_3_O_4_ in other forms, i.e., Ma et al. reported a specific capacitance of 130 F · g^-1^ (in 1 M Na_2_SO_4_ electrolyte at a current density of 1 A · g^-1^) for Mn_3_O_4_/graphene nanocomposites prepared by a one-step solvothermal process [29], and Wang et al. reported a specific capacitance of 159 F · g^-1^ (in 6 M KOH electrolyte at a scan rate of 5 mV · s^-1^) for Mn_3_O_4_/graphene synthesized by mixing graphene suspension in ethylene glycol with MnO_2_ organosol [30]. The high capacitance of the as-prepared Mn_3_O_4_/Ni foam composite can be attributed to the positive synergistic effects between Mn_3_O_4_ and Ni foam. The skeleton of Ni foam could reduce the aggregation of the Mn_3_O_4_ nanorods, making the Mn_3_O_4_ nanorod accessible for electronic and ionic transport pathways and enhancing the utilization of the active materials. Furthermore, Ni foam also provides a highly conductive network for electron transport during the charge and discharge processes.

The endurance test was conducted using galvanostatic charging-discharging cycles at 1 A · g^-1^ (insert of Figure 4d). The discharge capacitance loss after 2,000 consecutive cycles is about 20%. The specific capacitance degradation is estimated to be from 263 to 205 F · g^-1^ (Figure 4d). Although the Ni foam serves as a conductive matrix to promote fast Faradaic charging and discharging of the Mn_3_O_4_ nanorods, its loose structure leads to the flaking off of the nanorods from the Ni foam substrate.

### Time-dependent Mn_3_O_4_/Ni foam composite properties

**Figure 5:**
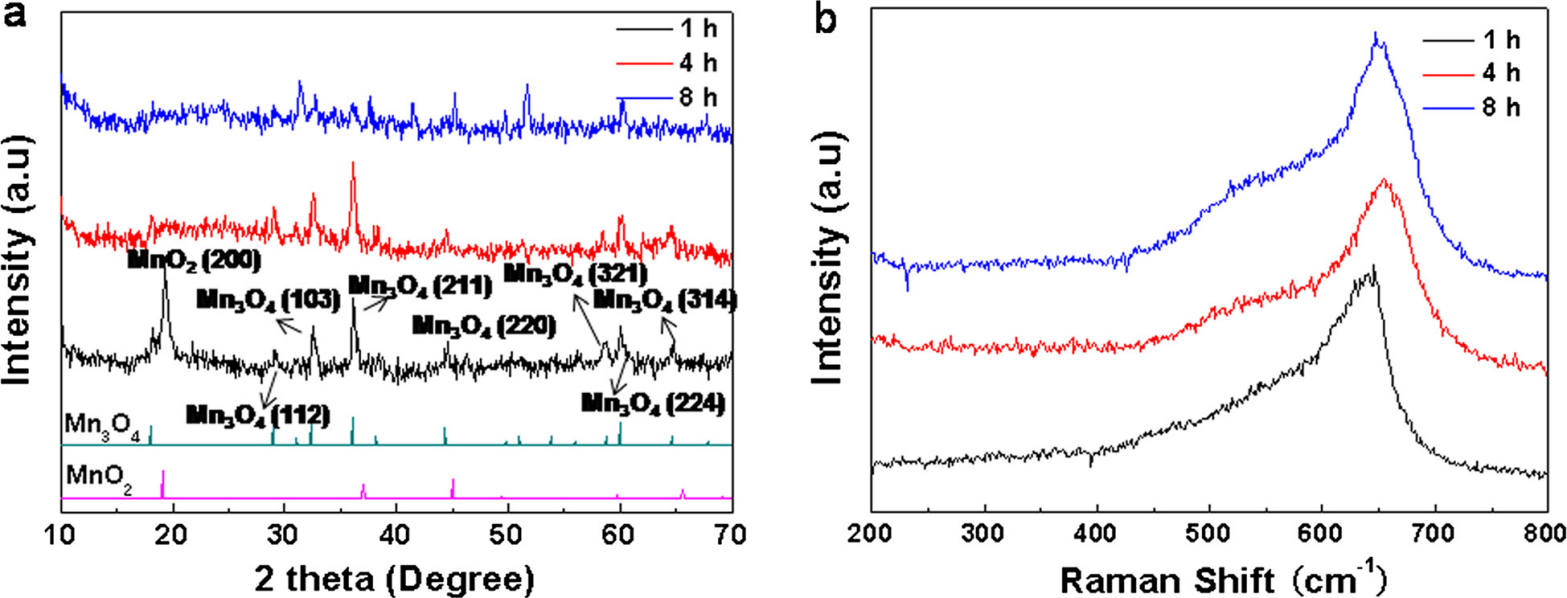
XRD pattern (a) and Raman spectra (b) of the powder scratched from composite electrode after different reaction time.

**Figure 6:**
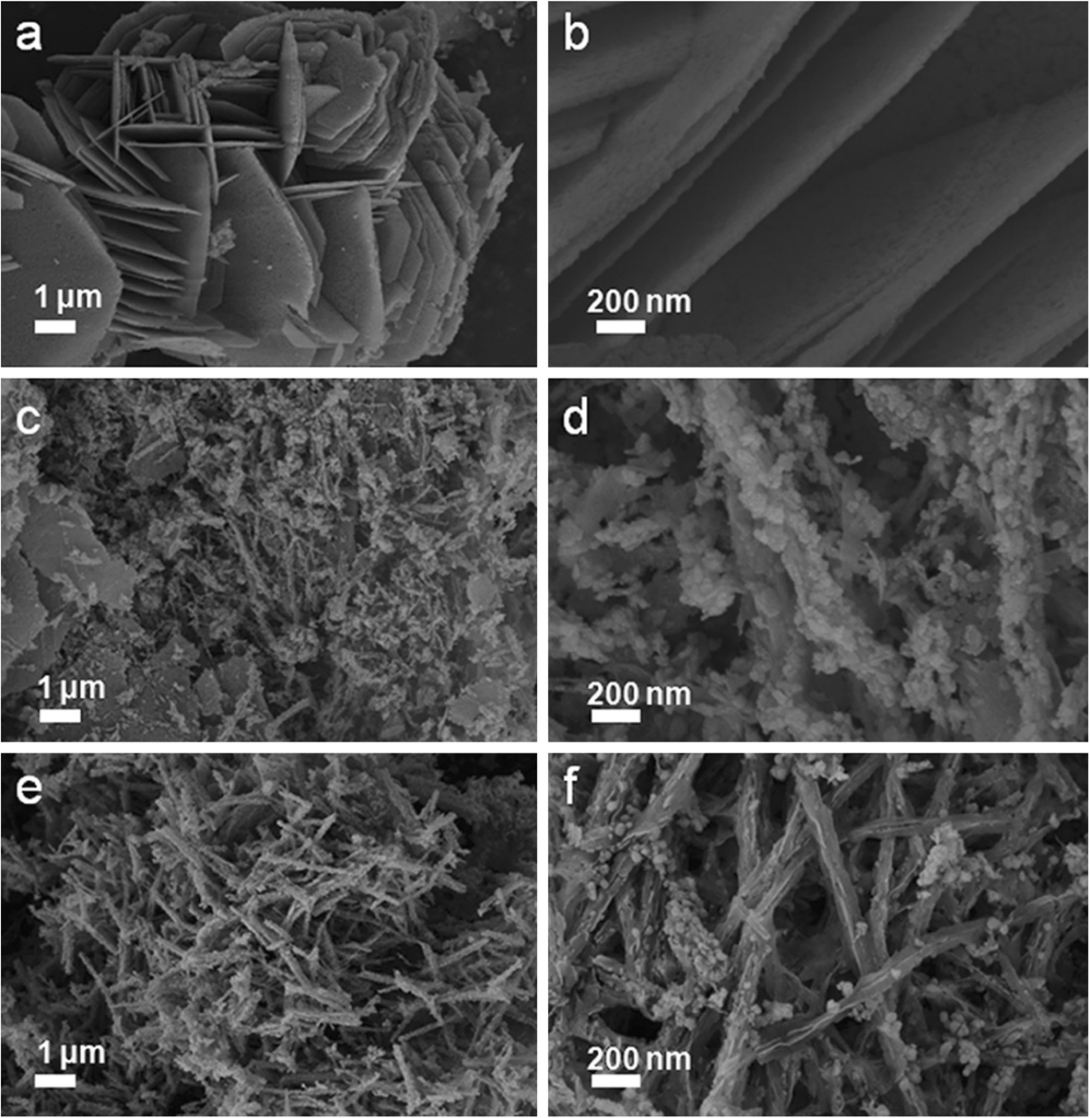
**SEM images of composite obtained after different reaction times. (a,b)** 1 h; **(c,d)** 4 h; **(e,f)** 8 h.

To shed light on the formation process, temporal evolution of the Mn_3_O_4_ nanostructures was studied by examining the products obtained under different reaction times of 1, 4, and 8 h. XRD patterns and Raman spectra were measured to identify the components of the different samples. The XRD patterns of the composite obtained under 1 h can be indexed to MnO_2_ and Mn_3_O_4_ crystal structures (Figure 5a). For the composites obtained under 4 and 8 h, the intense XRD peak at 2*θ* ≈ 19°disappeared corresponding to the MnO_2_ (200) crystal structures and the left peaks attribute to the Mn_3_O_4_ crystal structures. Figure 5b shows the Raman spectra of the powder scratched from composite electrodes. The peak position of composites obtained under 4 and 8 h are red shifted compared with that of the composite obtained under 1 h. As is known, the Raman spectra for the MnO_2_ phase and the Mn_3_O_4_ phase are located at 638.5 cm^-1^ and 652.5 cm^-1^, respectively [31]. Therefore, this red shift of Raman spectra indicates the component variation from the MnO_2_ phase to Mn_3_O_4_, which is in excellent agreement with the result obtained from the XRD study. The SEM images of products obtained under different reaction times of 1, 4, and 8 h are shown in Figure 6. The products collected after 1 h consisted of nanosheets with a thickness of about 30 nm (Figure 6a,b). When the reaction time increases to 4 h, some nanorods accompanied with nanoparticles begin to appear (Figure 6c,d). As the reaction proceeds to 8 h, the nanosheets disappeared and all of the products are nanorods with few nanoparticles (Figure 6e,f). After 10 h of the hydrothermal reaction, well-defined nanorods are obtained (Figure 3c,d). Based on the time-dependent morphology evolution described above, the formation mechanism of Mn_3_O_4_ nanorods can be proposed. At the initial stage, a large number of nanocrystallites nucleate and grow into nanosheets to minimize the overall energy of the system. However, the nanosheets are just intermediate products and not stable. After the reaction for 4 h, some of the nanosheets dissolve with the emergence of nanorods with some nanoparticles. When the reaction proceeds for 8 h, all of the nanosheets have transformed into nanorods with nanoparticles. This suggests that the dissolution of nanosheets and the growth of nanorods occur simultaneously during the hydrothermal reaction. Further increase of the reaction time results in the development of well-defined and uniform nanorods without any impurity.

**Figure 7:**
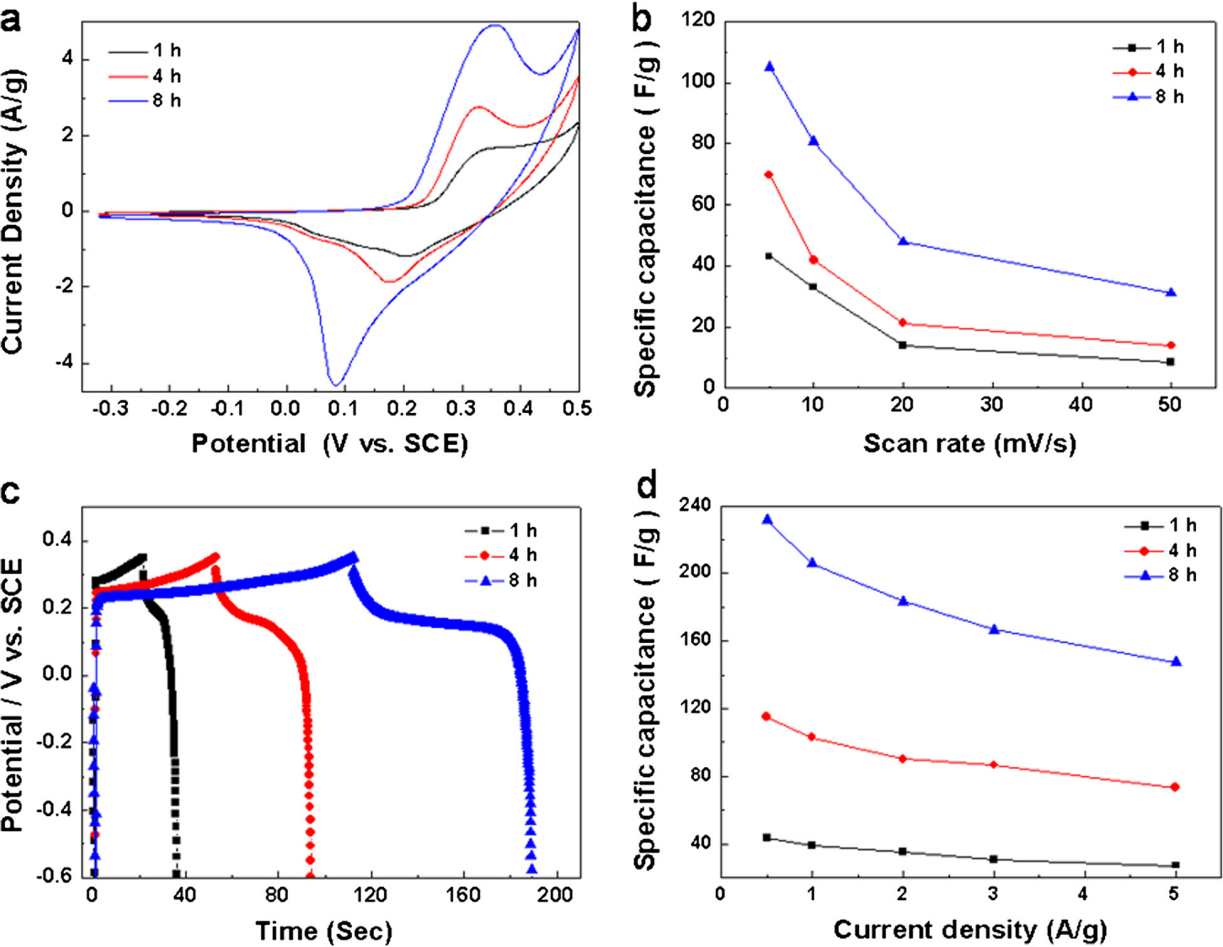
**Supercapacitive properties of composite obtained after different reaction times (1, 4, and 8 h). (a)** CV curves recorded in 4 M NaOH solution at 20 mV · s^-1^; **(b)** corresponding specific capacitance as a function of scan rate; **(c)** charging-discharging curves at 1 A · g^-1^current density; **(d)** corresponding specific capacitance as a function of current density.

The electrochemical properties of products obtained under different reaction time were studied in 4 M NaOH solution. Figure 7a shows the CV curves of the products at a scan rate of 20 mV · s^-1^. As the reaction time increases from 1 to 8 h, the redox current density increases. The product obtained under 8 h may show the best capacitive behavior of the three products because the specific capacitance increases with the current density at the same scan rate. Figure 7b depicts the specific capacitance of the products under different reaction time at scan rates between 5 and 50 mV · s^-1^. All of them show that the specific capacitance gradually decreases as the scan rate increases, which can be attributed to the diffusion limitations in pore [22]. Obviously, the product obtained at 8 h has the highest specific capacitance, consistent with the CV tests in Figure 7a. The discharge curve of the composite obtained under 8 h displays a longer plateau than that of 1 and 4 h at 1 A · g^-1^ (Figure 7c). It is known that the increase of the charging time represents the higher capacitance at a fixed discharge current density. The dependence of the specific capacitance on the current density is compared in Figure 7d. The specific capacitance of the composite obtained at 1 h is 44, 39, 35, 31, and 27 F · g^-1^ at 0.5, 1, 2, 3, and 5 A · g^-1^, respectively. For current densities beyond 5 A · g^-1^, the iR drop is too large to permit an accurate calculation of the specific capacitance. In contrast, the specific capacitance of the composite obtained at 8 h is 232, 206, 183, 167, and 147 F · g^-1^ at the corresponding current densities. Combined with the curve in Figure 4b, the composite obtained at 10 h exhibits the highest specific capacitance. The increase in the specific capacitance can be attributed to the unique structure of the composite, and a longer period of reaction time leads to closer contact between the Ni foam substrate and the active material. Similar phenomena were also observed at the nanostructured Ni(OH)_2_/Ni foam whose specific capacitance reached the highest after the longest reaction time [32].

### Electrochemical capacitance of Mn_3_O_4_/Ni plate electrode- comparison with Mn_3_O_4_/Ni foam

**Figure 8:**
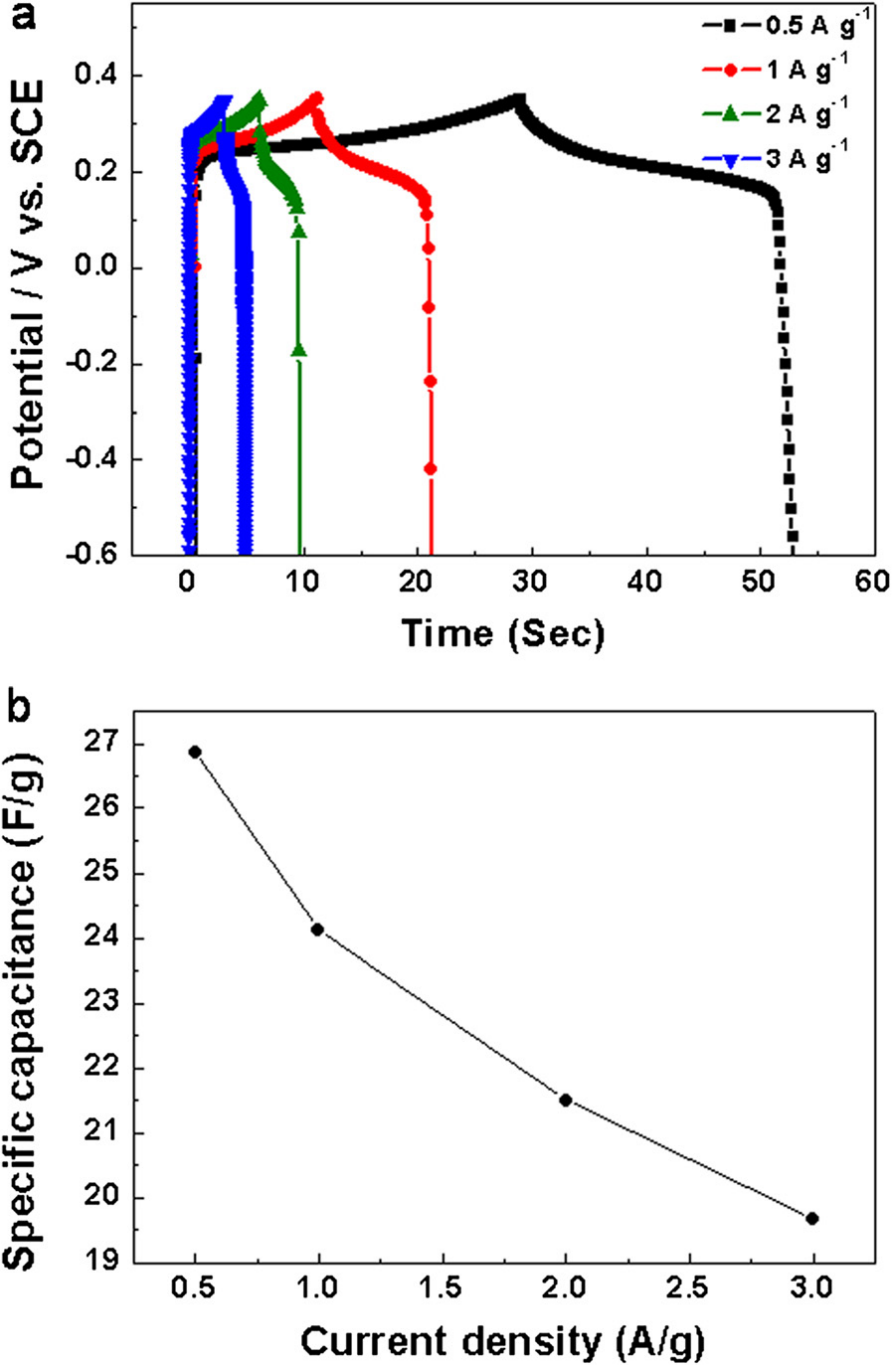
**Charging-discharging curves of Mn**_**3**_**O**_**4**_**/Ni plate electrode (a) and corresponding specific capacitancesas a function of current density (b). (a)** Curves are measured at different current densities.

As is known, the substrate is important to pseudocapacitor electrode materials. To investigate the electrochemical capacitance of the composite as a function of the substrate, control experiment was conducted using the Ni plate instead of the Ni foam for Mn_3_O_4_ growth under the same condition. Figure 8a shows the charging-discharging curves of the Mn_3_O_4_/Ni plate measured at different current densities. Compared with the curve in Figure 4b, the decrease in the charging time represents the lower capacitance of the Mn_3_O_4_/Ni plate. The specific capacitances of the Mn_3_O_4_/Ni plate are 27, 24, 21, and 19.6 F · g^-1^ at 0.5, 1, 2, and 3 A · g^-1^, respectively (Figure 8b). The specific capacitance of the Mn_3_O_4_/Ni foam is more than 10 times higher than that of the Mn_3_O_4_/Ni plate. The Ni foam substrate with microholes and zigzag flow channels results in excellent mass transport property and large surface area per unit volume of the electrode.

## Conclusions

A facile one-step hydrothermal method was successfully developed to synthesize Mn_3_O_4_ nanorods on Ni foam. The complete absence of any surfactant enabled the product to have high purity. The formation process was proposed to include the dissolution of nanosheets, followed by the formation of uniform nanorods. The obtained Mn_3_O_4_ nanorods have diameters of about 100 nm and lengths of 2 to 3 μm. A high specific capacitance of 263 F · g^-1^ has been achieved for the Mn_3_O_4_/Ni foam at 1 A · g^-1^, which is higher than that of the Mn_3_O_4_ composite on other substrates. Porosity may enhance the electrolyte/Mn_3_O_4_ contact area and shorten the electrolyte diffusion length in the nanostructures. The cost-effective fabrication and remarkably high specific capacitance provide great potential for this type of hybrid nanostructure to be used as an active electrode for supercapacitor application.

## Competing interests

The authors declare that they have no competing interests.

## Authors' contributions

YZ and DL designed this research. DL carried out the experiments and analyzed the data. FM, XY, LY, and HH contributed to the discussion. DL and YZ wrote the paper. All authors read and approved the final manuscript.
